# Utilization of long-acting and permanent contraceptive methods and associated factors among married women in Adama town, Central Ethiopia: community based cross-sectional study

**DOI:** 10.1186/s40834-019-0101-5

**Published:** 2019-12-09

**Authors:** Markos Desalegn, Ayele Belachew, Muluken Gizaw, Gemechu Kejela, Robsan Gudeta

**Affiliations:** 1 Department of Public health, College of medicine and health sciences, Jigjiga University, Jigjiga, Somali region Ethiopia; 2 School of public health, college of health sciences, Addis Ababa University, Addis Abeba, Ethiopia; 3 Department of public health, Institute of health sciences, Wollega University, Oromia region, Nekemte, Ethiopia

**Keywords:** Utilization, Long acting and permanent, Contraceptive methods

## Abstract

**Background:**

Long-acting and permanent contraceptive methods have clear advantages over short-acting methods of contraception that benefit both clients and health systems. Despite this importance, studies show that the proportion of women currently using long acting and permanent contraceptive methods are significantly lower than the proportion using short-acting methods.

**Objective:**

The main aim of the study was to assess the level of utilization of long acting and permanent contraceptive methods and associated factors among married women in Adama town.

**Methodology:**

Community Based Cross-Sectional Study was conducted in four kebeles of Adama town from April 15–30, 2015. Multistage sampling technique was used to select the study participants. The collected data was cleaned and entered using Epi info 3.5.3 and analyzed using statistical package for social science version 20.0. Factors associated with utilization of long acting and permanent contraceptive methods were identified using logistic regression model.

**Result:**

In this study, the magnitude of long acting and permanent contraceptive methods was 20.9%. Implant, Intra-Uterine devices (IUDs) and tubal ligation accounted for 16.1, 4.6, and 0.2% respectively. Current use of long acting and permanent contraceptive methods was higher among women who had high knowledge (AOR = 5.26, 95% CI = 1.90–14.69), positive attitude (AOR = 3.25, 95% CI = 1.60–6.58) and women who had 3–4 children (AOR [95%CI] =2.3[1.14–4.63]) compared to those who had no child.

**Conclusion:**

Current use of long acting and permanent contraceptive methods in Adama town was low. Level of knowledge, attitude about the methods, and number of children were factors affecting utilization of long acting and permanent contraceptive methods. Targeted Information Education Communication Intervention should be intensified to improve the utilization of these methods.

## Background

Family planning is a means by which individuals or couples space pregnancy and childbirth at intervals, mutually determined by both husband and wife in order to have the desired number of children [1].

Four contraceptive methods are categorized as Long-Acting and Permanent Contraceptive Methods (LAPCMs): Intra-Uterine Devices (IUDs), Implants, Tubal ligation, and Vasectomy. IUDs and Implants are long-acting methods; when removed, return to fertility is prompt. Copper-containing IUDs are effective for 10–12 years. Implants, depending on the type, last for 3–5 years. Tubal ligation and Vasectomy are permanent methods [2, 3].

LAPCMs have clear advantages over short-acting methods of contraception that benefit both clients and health systems. It increases the contraceptive method options that couples have, especially as their need evolve over time and is the best choice for couples both to space and limit childbirth and is the best way to protect women and couples against unwanted pregnancies [4].

Over time, the use of Long-acting and permanent contraceptive methods has not kept in pace with that of short-acting methods, such as oral contraceptives and injectables [5]. Surveys from four Sub-Saharan countries showed that, the proportion of women currently using LAPCMs is significantly lower than the proportion using short-acting methods, which accounts for less than 5 % [6]. Like other Sub-Saharan countries, Long-Acting and Permanent Contraceptive Methods use have lowest rates in Ethiopia (4.2%) for which IUD, Implant and Female sterilization contribute 0.3,3.4 and 0.5% respectively [7, 8].

The growing use of contraception around the world has given couples the ability to choose the number and spacing of their children and has had tremendous lifesaving benefits [9]. Despite this, approximately 25% of Sub-Saharan African couples and 29% of Ethiopian couples, who need to space or limit births, are not using any form of modern contraception [7, 8].

Therefore, the aim of this study was to assess the utilization of LAPCMs and associated factors among married women.

## Methods and materials

### Study area

The study was conducted in Adama town, located to the east of Addis Ababa at the distance of 99 km. The town has 20 kebeles (the smallest administrative unit in Ethiopia with a geographical boundary of having a minimum of 1000 households). According to the 2007 Census conducted by the Central Statistical Agency of Ethiopia (CSA), the city has a total population of 220,212. About 60,174 households were counted in the city, which results in an average of 3.66 persons to a household, and 59,431 housing units [10]. Adama town has one government hospital, three private hospitals; six government health centers, one private clinic, one maternal health clinic (Marie Stopes), one Family Guidance Association of Ethiopia (FGAE) and 72 drug shops. From these health institutions, family planning service is being delivered in all hospitals, health centers, FGAE, and Marie Stopes clinics.

### Study period

The study was conducted from April 15–30, 2015.

### Study design

Community-based cross-sectional survey was conducted to assess the utilization of LAPCMs and associated factors among married women aged 15–49 in Adama town.

### Source population

The source populations of the study were all married women aged 15–49 years who live in Adama town during the study period.

### Study subject

The study subject in this study was a sampled married women of age 15–49 years who live in Adama town during the study period.

### Sample size determination

To estimate the sample size, a single population proportion formula with the following assumptions was used.

$$ n=\frac{{\left( Z\alpha /2\right)}^2P\ \Big(1-(P)}{d^2} $$

### Assumptions

Desired precision (d) = 5%.

Proportion of long acting and permanent contraceptive methods clients from the previous study done in Adigrat and Debre Markos town was 19.5% each [11, 12].

Confidence level = 95%, which means α set at 0.05 and Ζα/2 = 1.96 (value of Ζ at α 0.05 or critical value for normal distribution at 95% CI [13].

$$ n=\frac{{\left( Z\alpha /2\right)}^2P\ \Big(1-(P)}{d^2} $$

$$ {\displaystyle \begin{array}{c}n=\frac{(1.96)^20.195\Big(1-(0.195)}{(0.05)^2}\\ {}=241\end{array}} $$

Using a design effect of 2(since it is a multistage sampling) and a 10% non-response rate, the calculated minimum sample size was 530.

### Sampling procedures

A multi-stage sampling technique was used to select the study participants. The town has 20 kebeles. Out of this, four kebeles were selected using simple random sampling. Then, the number of households with reproductive-age women in each selected kebele were identified and proportionally allocated for the sample size. Sampling fraction was calculated for each kebele and initial household with eligible married women was picked randomly. Finally, study samples were selected using a systematic sampling method. Only one woman was interviewed for households having two or more married women on random to avoid intra-class correlation [13]. In the absence of married women in the sampled household, interviewer jumped to the next house.

### Inclusion and exclusion criteria

Married women of reproductive age group (15–49), women who have lived in the town for more than six months were included. Women having a serious illness and mental disorder were excluded.

### Data collection and questionnaire

A structured interviewer-administered questionnaire was used. The questionnaire was adopted from similar studies done in Mekelle and Adigrat town [11, 13]. It was modified based on the situation of the study to collect information on the utilization of long acting and permanent contraceptive methods and associated factors from married women in Adama town. The questionnaire was prepared in English, translated to Amharic language and translated back to English by another person in order to check its consistency.

The questionnaire has five sections: socio- demography (9 items), reproductive history (6 items), knowledge (18 items), attitude (10 items) and practice of modern contraception (12 items).

The questionnaire was pretested before actual data collection among 28 respondents (5% of the calculated sample size) in Modjo town. Because population residing in Modjo town share almost similar characteristics with the population in the study area.

Five health extension workers were trained for data collection on the content of the questionnaire and approach to participants during data collection. They were selected based on their ability to speak and write Afan Oromo and Amharic languages.

During data collection, one supervisor was selected to supervise the data collector’s activity, checking the completeness of the questionnaire and receive the collected and completed questionnaire. Participants were interviewed, where they were free to express their idea freely. Moreover, on occasions when the sampled women were not be accessed for absence, up to two attempts was endeavored for interviewing to lessen the non-response rate.

### Data processing and analysis

Data were coded, entered into Epi info version 3.5.3 and exported to SPSS version 20.0 for analysis. Descriptive statistics were computed to determine the frequency and percentages. Binary logistic regression was conducted and COR, with 95% CI was estimated to select the candidate variables for the final model. Then, variables with a *p*-value of < 0.2 at binary logistic regression were taken into a multivariable logistic regression to control confounding. Hosmer-Lemeshow goodness-of-fit with stepwise (backward elimination) logistic regression was used to test for model fitness. AOR with 95% CI was estimated to assess the presence of association at multivariable logistic regression. Finally, variables with a *p*-value of < 0.05 were considered as statistically significant predictors of the outcome variable.

### Operational definition

#### Utilization of LAPCMs

Is the current use of contraceptive implants or IUDs or Tubal Ligation by the women (study participant) or Vasectomy (male sterilization) by her husband.

#### User of LAPCMs

Women who were using either contraceptive implants or IUDs or Tubal Ligation by herself or Vasectomy (male sterilization) by her husband during the data collection period.

#### Non-user of LACMs

Were women who were not using contraceptive implants, either IUDs or Tubal Ligation by herself or Vasectomy (male sterilization) by her husband during the data collection period.

#### Attitude of LAPCMs

Refers to one’s own opinion, belief and perception about LAPCMs. In this study, the attitude of the respondent was measured by attitude statements like using Implant do not lead to abnormal bleeding, Implant and IUD do not move/escape in the body after insertion and using LAPCMs do not lead to abnormal bleeding. It was labeled as a positive and negative attitude where those who have scored above mean of attitude questions was grouped as a positive attitude and mean or below mean was grouped as negative attitude [13].

#### Knowledge of LAPCMs

Refers to one’s own awareness and familiarity with LAPCMs. Ten knowledge questions were used to grade the knowledge of LAPCMs. It was graded as low knowledge; those who have answered less than 60% of knowledge question; Moderate knowledge; those who have answered 60–79% of knowledge question; High knowledge: those who have answered above or 80% of knowledge question [13].

#### Kebele

is the smallest administrative unit in Ethiopia with a geographical boundary of having a minimum of 1000 households.

#### Zone

Is small administrative subdivision within the kebele.

### Data quality management

To assure the quality of the data, structured interviewer-administered questionnaire was used to collect information. Before the actual data collection, pre-test had been conducted. Data collectors were trained for one day and they have been informed about how to approach the respondents, how to apply the designed data collection method, how to ask each of the questions, follow the instructions of the questionnaire and to keep the confidentiality of the respondents. One supervisor was assigned to check the completeness of the questionnaire every night with the principal investigators.

## Results

### Socio-demographic characteristics of the respondents

Five hundred twenty-six married women were interviewed, making response rate of 99.25%. The mean age of the respondents was 29 years (+SD 6.31), majority 297(56.5%) of which belong to the age group of 25–34. Many of the participants were Oromo 220 (41.8%) in ethnicity (data not shown in the table). More than half of the respondents were Orthodox 297 (56.5%) in religion. Most of the married women interviewed were educated 477(92.2%), whereas by occupation, 127 (51.5%) of the participants were homemaker. The mean family size of the respondents was 4 (3.9 + SD). The majority, 246 (46.8%) of the study respondents have family sizes of 2–3 (Table 1).

**Table 1:** Socio-demographic characteristics of the study participants, Adama town, April 2015

S/No	Socio demography	frequency	Percentage
1	Age		
	15–24	128	24.3
	25–34	297	56.5
	35–44	87	16.5
	> 45	14	2.7
2	Religion		
	Orthodox	297	56.5
	Muslim	130	24.7
	Protestant	99	16.9
	Others*	10	1.9
3	Educational status		
	No education	49	9.3
	Only read and write	30	5.7
	Elementary [1–8]	161	30.6
	Secondary [9, 10]	107	20.3
	Senior secondary [11, 12]	66	12.5
	Diploma and above	113	21.5
4	Family size		
	2–3	246	46.8
	4–5	213	40.5
	> 5	67	12.7

Others*: catholic and Adventist

### Reproductive characteristics of the participants

About 151 (34.7%) of the respondents did not need to have more children and 236 (54.3%) of the participants need to have 1–2 more children. From all participant, 234(44.6%) want to have a child in the next two years and 292 (55.4%) did not want to have a child in the next two years (Table 2).

**Table 2:** Reproductive history of the study participants, Adama town, April 2015

S/No	Reproductive history	Frequency	Percentage
1	Ever gave birth (*n* = 526)		
	yes	435	82.7
	No	91	17.3
2	Number of birth (n = 435)		
	1–2	283	65.1
	3–4	117	26.9
	> 4	35	8
3	Number of living children (*n* = 435)		
	0	2	0.5
	1–2	293	67.4
	3–4	110	25.3
	> 4	30	6.9
4	An additional number of the child they need (n = 435)		
	0	151	34.7
	1–2	236	54.3
	> 2	48	11
5	Discussion about modern contraception (n = 526)		
	Yes	474	90.1
	No	52	9.9

### Knowledge of the participants about long acting and permanent contraceptive methods

From the total respondents, 512(97.3%) have heard at least one modern contraceptive methods. About 479 (93.6%), 508 (99.2%), 433 (84.6%), 130 (25.4%) and 76 (14.8%) of respondents have heard Pill, Injectable, Implant, Tubal ligation and Vasectomy as contraceptive methods respectively. The major source of information was the health profession 453(88.5%) followed by mass media 423(82.6%).

Four hundred thirty-five (87%) of the respondents knew about long acting and permanent contraceptive methods**.** Majority of them (66.3%) knew that IUD prevents pregnancy for more than 10 years. About 69 and 72.1% knew that pregnancy is possible immediately after removal of IUD and implant respectively. Eighty-seven percent of them had awareness of LAPCMs; 63.7% as it prevents possible child and maternal death; 69.5% as it spaces childbirth and 91% as it limits the family size. Concerning the level of knowledge, 46.8% of the participant had high knowledge, 24.5% had moderate knowledge and 28.7% had low knowledge (Tables 3 and 4).

**Table 3:** knowledge of modern contraceptive methods among study participants

S/No	Variables	Frequency	Percentage
1	Ever heard of modern contraceptive method (n = 526)		
	Yes	512	97.3
	No	14	2.7
2	Type of contraceptive method known (*n* = 512)		
	Pill	479	93.6
	Injectable	508	99.2
	Implant	433	84.6
	IUD	130	25.4
	Tubal ligation	76	14.8
	Vasectomy	368	71.9
	Condom	388	71.9
3	Source of information(n = 512)		
	Friend, neighbor, and relatives	137	26.8
	Husband	54	10.5
	Health professionals	453	88.5
	Mass media	423	82.6

Each of the percentages does not add up to 100.00 because respondents could choose several responses, which could be spontaneous or prompted

**Table 4:** knowledge level of the study participants, Adama town, April 2015

	Knowledge variables (n = 512)	knowledge level			
		True		False	
		Frequency	percent	frequency	Percent
1	IUD prevent pregnancy for > 10 years	404	78.9	108	21.1
2	IUD is not good for women at risk of STI	246	48	266	52
3	IUD has no effect on sexual desire	349	68.2	163	31.8
4	Pregnancy is possible after the removal of IUD	362	70.7	150	29.3
5	Implant prevent pregnancy for > 5 years	423	82.6	89	17.4
6	Implant has no effect on sexual desire	409	79.9	103	20.1
7	Pregnancy is immediate after removal of the implant	379	74	133	26
8	Vasectomy has no effect on sexual desire	205	40	307	60
9	Pregnancy is not possible after tubal ligation	312	60.9	200	39.1

### The attitude of participants about long acting and permanent contraceptive methods

Only 137 (26%) of the participant agreed that Implant does not bring about excessive bleeding. Three hundred thirteen (59.6%) of them perceived that insertion of IUD exposes once own privacy and 432 (82.3%) perceived that IUD block ordinary activities. Four hundred fifty-eight (87%) of the participants perceived that Implant moves and escapes in the body while most of them perceive that insertion and removal of the Implant is pain full. Concerning the respondent’s general attitude status, 55.1% of the participant had a positive attitude towards LAPCMs while the rest 44.9% had a negative attitude (Table 5).

**Table 5:** Attitude of study participants about LAPCMs, Adama town, April 2015

S/No	Perception statement(*n* = 525)	Level of agreement					
		Agree		Not sure		Disagree	
		frequency	Percent	frequency	percent	frequency	percent
1	Implant do not lead to bleeding	137	26.1	162	30.9	226	43
2	Insertion of IUD does not lead to loss of privacy	212	40.4	172	32.8	141	26.8
3	IUD does not move through the body	85	16.2	163	31	277	52.8
4	IUD do not prevent ordinary activity	93	17.7	134	25.5	298	56.8
5	IUD do not lead to excessive bleeding (*n* = 524)	93	17.7	183	35	248	47.3
6	Operation of Tubal ligation is not dangerous	50	9.5	307	58.5	168	32
7	Insertion and removal of implant is painful	52	9.9	165	31.4	308	58.7
8	Implant do not move through the body	67	12.8	127	24.2	331	63
9	spouse cannot decide on wife’s FPM choice	253	48.2	36	6.8	236	45
10	HP cannot decide on method choice of FP.	142	27	19	3.7	364	69.3

### Utilization of long-acting and permanent contraceptive methods among respondents

#### Ever use of modern contraceptive methods

The ever use of the modern contraceptive methods in this study area was 87.1%. The most ever used method was Injectable (66.3%).

#### Current use of modern contraceptive methods

Current modern contraceptive use was 59.8%. The most preferred method was Injectable 159(30.1%) and Implants 85(16.1%). The rest were using Pill 41(7.8%), IUD 24(4.6%), Condom 5(1%) and Tubal ligation 1(0.2%).

#### None use of modern contraceptive methods and the reasons for nonuse

**Fig. 1:**
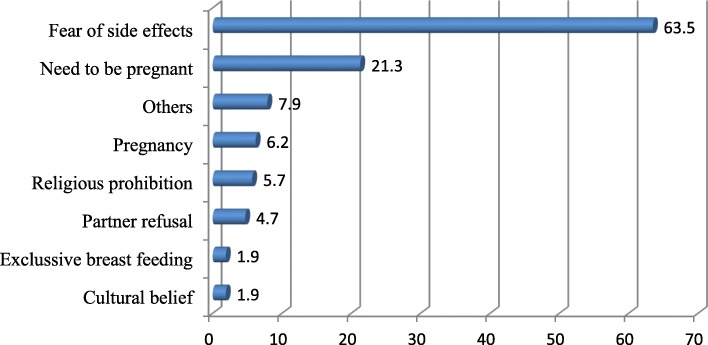
Reason for not using modern contraceptive method among study participants, Adama tow, April 2015

About 40.2% of the respondents were not using any modern contraceptive methods (Table 6). Sixty-three point 5 % (63.5%) of them reported that they were not using modern contraceptive methods because of fear side effects (Fig. 1).

**Table 6:** Utilization, the reason for not using and service center of modern contraceptive methods of the study participants, Adama town, April 2015

S/No	Variables	Frequency	Percentage
1	Current use of methods (*n* = 315)		
	pill	41	7.8
	Injectables	159	30.1
	Implant	85	16.1
	IUD	24	4.6
	Tubal ligation	1	0.2
	Condom	5	1
3	Service center (n = 315)		
	Health center	165	52.4
	Hospitals	51	16.2
	Private clinic	60	19.0
	Pharmacy	4	1.3
	HEW	3	1.0
	Relatives	1	0.3
	Others****	31	9.8

Others****: Mariestops and FGAE

The overall prevalence of the LAPCMs was 20.9%. Majority of them use Implant (16.1%) and IUD (4.6%). The rest 0.2% use tubal ligation. Health center was where majority of the users get the service (52.4%) while others get service from other government organization (16.2%) and private clinic (19%).

Utilization of LAPCMs among married women who had no formal education was 2 and 28.3% among diploma and above. Current use of LAPCMs was 4.4% among married women who did not give birth. However, 37.6 and 25.7% of married women who gave birth to 3–4 and > 4 children were using LAPCMs respectively.

**Fig. 2:**
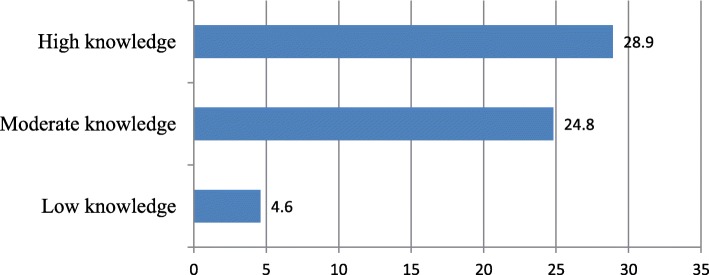
Current use of LAPCMs by knowledge level of the study participants, Adama town, April 2015

**Fig. 3:**
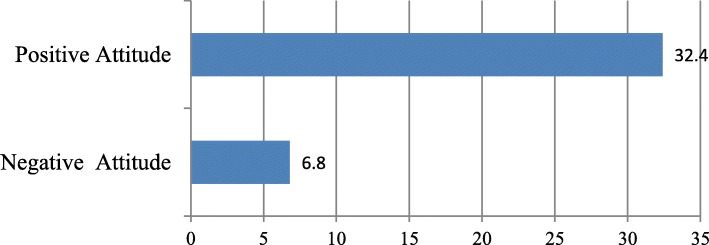
Current Use of LAPCMs by Attitude status of the study participants, Adama town, April 2015

Current use of LAPCMs among those with low knowledge and negative attitude was, 4.6 and 6.8% respectively. However, current use among those with high knowledge and positive attitude was 28.9 and 32.4% respectively (Figs. 2 and 3).

### Factors associated with utilization of long-acting and permanent contraceptive methods

The result of multivariate analysis revealed that knowledge level was one of the predictors of the current use of LAPCMs. Married women with high knowledge use LAPCMs 5 times more likely than those with low knowledge (AOR [95% CI] = 5.26[1.90–14.69]), and those with moderate knowledge use 3 times more likely compared to those with low knowledge (AOR [95%CI] = 3.27[1.12–9.52]). The attitude of the respondent was also known to predict the current use of LAPCMs in which those who had a positive attitude about LAPCMs use the methods 3 times more likely compared to those who had a negative attitude (AOR [95%CI] =3.25[1.60–6.58]). In this model, married women who gave birth to 3–4 children use LAPCMs 2 times more likely compared to those who had given birth to 1–2 children (AOR [95%CI] = 2.3[1.14–4.63]). Participants who need to have 1–2 more children had 67% lesser odds of using LAPCMs compared to a participant who didn’t need any more children (AOR [95%CI] = 0.33[0.16–0.66]) (Table 7).

**Table 7:** Multivariate and Bivariate analysis of selected variables affecting utilization of LAPCMs among married women aged 15–49 years, Adama town, April 2015

Variables	Total	Utilization of LAPCMs			
		Yes	No	COR,(95% CI)	AOR,(95 CI)
Age of married women					
15–24	66	9 (13.6)	57 (86.4)	1.0(ref)	
25–34	269	64 (23.8)	205 (76.2)	2,(0.93–4.22)	1.43,(0.57–3.60)
35–44	87	33 (37.9)	54 (62.1)	3.87,(1.70–8.84)	1.60,(0.51–5.00)
> 45	13	2 (15.4)	11 (84.6)	1.15,(0.22–6.07)	1.13,(0.14–9.13)
Occupation of women					
Housewife	235	53 (22.6)	182 (77.4)	1.0(ref)	
Gov’t employee	76	26 (34.2)	120 (78.9)	1.79 (1.02–3.14)	0.90,(0.42–1.94)
Daily labourer	39	4 (10.3)	35 (89.7)	0.4 (0.13–1.15)	0.34,(0.10–1.16)
Merchant	53	14 (26.4)	39 (73.6)	1.24 (0.62–2.44)	0.84 (0.36–1.94)
Student	18	4 (22.2)	14 (77.8)	0.98 (0.3–3.10)	0.76 (0.18–3.18)
NG0s	14	7 (50)	7 (50)	3.4 (1.15–10.2)	4,(1.04–16.20)*
No of birth					
1–2	283	53 (19.4)	230 (80.6)	1.0(ref)	
3–4	117	44 (37.6)	73 (62.4)	2.5,(1.55–4.02)	2.30,(1.14–4.63)*
> 4	35	9 (25.7)	26 (74.3)	1.44,(0.64–3.24)	1.43,(0.35–5.92)
Knowledge level					
Low knowledge	123	7 (5.7)	144 (94.3)	1.0(ref)	
High knowledge	202	69 (34.2)	133 (65.8)	8.6 (3.8–19.45)	5.26 (1.9–14.6)*
Moderate knowledge	110	32 (29.1)	78 (70.9)	6.8 (2.86–16.17)	3.27 (1.12–9.52*
Attitude status					
Negative attitude	187	16 (8.6)	171 (91.4)	1.0(ref)	1
Positive attitude	248	92 (37.1)	156 (62.9)	6.3 (3.55–11.18)	3.25 (1.60–6.58)*
Number of the child they wanted					
0	151	59 (39.1)	92 (60.9)	1.0(ref)	1
1–2	236	40 (16.9)	196 (83.1)	0.32 (0.20–0.50)	0.33 (0.16–0.66)*
> 2	48	9 (18.8)	39 (81.2)	0.36 (0.16–0.80)	0.5 (0.17–1.48)

*Respective variables are statistically significant

## Discussions

The magnitude of the current use of LAPCMs in this study was 20.9%. This result was in line with studies conducted in Adigrat and Debra Markos (19.5%) each [11, 12]. The most preferred method was Implant (16.1%) followed by IUD (4.6%) and Tubal ligation (0.2%). This finding was greater than the study conducted in Nigeria (24.7%) [14].

In multivariate analysis, knowledge level, attitude status, and a number of children were found to affect the current use of LAPCMs among respondents.

Participants who had high knowledge and moderate knowledge used LAPCMs five and three times more likely compared to the participant of low knowledge. Similarly, a study done in Mekelle revealed, participant of high knowledge had used LAPCMs 8 times more likely compared to low knowledge level. Those who had moderate knowledge used LAPCMs 3 times more likely compared to the participant of low knowledge [13]. In addition, finding in Uganda revealed, knowing the duration of protection of IUD and Implant was positively associated with the use of LAPCMs [15]. EDHS 2011 showed, even knowing at least one contraceptive method was prerequisite for use of a contraceptive method [8]. This might be due to the knowledge of contraceptive method helps women to know what and where to use and get the method.

Current use of LAPCMs was higher among participants who had a positive attitude about LAPCMs than those who had a negative attitude. This finding was similar to other findings in Ethiopia where women with positive perceptions about contraceptive methods used contraceptive methods more likely [16, 17]. In Malaysia practice of LAPMs was surrounded by fear and misperception about a modern contraceptive method like IUD that it had to harmful effects on the health [18]. This might be due to, having a positive attitude was prerequisite for using contraceptive method [8, 17].

Participants who gave birth to 3–4 children used LAPCMs more likely than participants who did not give birth. This finding was almost in line with finding in Mekelle where mothers who had more than two pregnancy use LAPCMs three times more likely than mothers who didn’t experience any pregnancy [13]. This might be due to fear of infertility after removal of LAPCMs. Participants who need to have more children [1, 2] had 67% lesser odds of using LAPCMs. This might be due to a significant amount of the respondents in this study did not know that pregnancy is immediate after removal of IUD (31.2%) and Implants (27.9%).

## Conclusion and recommendation

The study showed that the utilization of LAPCMs is low despite several efforts made by the federal ministry of health and other stakeholders to escalate it. The main reason for not using the method was the fear of side effects. Knowledge level, attitude status, and parity were found to be associated with utilization of LAPCMs. Information education communication should be intensified and further research should be conducted to explore for other factors associated with utilization of LAPCMs.

## Abbreviations

ACIPH: Addis Continental Institute of Public Health
BRDC: Birhan Research and Development Consultancy
CPR: Contraceptive Prevalence Rate
CSA: Central Statistical Agency
EDHS: Ethiopian Demographic Health Survey
FGAE: Family Guidance Association of Ethiopia
FGD: Focus Group Discussion
FMOH: Federal Ministry of Health
FP: Family Planning
HEW: Health Extension Workers
IDHS: Indonesia Demographic Health Survey
IUD: Intra-Uterine Device
KAP: Knowledge, Attitude and Practice
LACMs: Long-Acting Contraceptive Method
LAPCMs: Long-Acting and Permanent Contraceptive Methods
MSI: Marie Stopes International
NPC: National Population Commission
OCs: Oral Contraceptives
PMs: Permanent Methods
SNNP: Southern Nation Nationality and Peoples
TFR: Total Fertility Rate
USAID: United State Agency for International Development
WHO: World Health Organization
